# Development and validation of a machine learning-based risk prediction model for PICC-associated bloodstream infections in preterm infants: a retrospective multicenter study

**DOI:** 10.3389/fped.2026.1838914

**Published:** 2026-08-13

**Authors:** Ruiqing Song, Zhirui Li, Denghui Ma, Xiaomin Yin, Lingxi Li, Junge Li, Kun Wang

**Affiliations:** 1 Department of Neonatal Intensive Care, The Third Affiliated Hospital of Zhengzhou University, Zhengzhou, China; 2 Department of Nursing, The Third Affiliated Hospital of Zhengzhou University, Zhengzhou, China

**Keywords:** catheter-related bloodstream infection, machine learning, peripherally inserted central catheter, preterm infants, risk prediction

## Abstract

**Objective:**

To develop and validate a machine learning–based model for predicting the risk of peripherally inserted central catheter (PICC)–associated bloodstream infection (CRBSI) in preterm infants.

**Methods:**

This retrospective multicenter study included 151 preterm infants with CRBSI and 302 matched controls from a tertiary hospital (2017–2024), randomly divided into a training set (*n* = 317) and an internal validation set (*n* = 136). An additional 96 cases from four tertiary hospitals were used for external validation. Eight significant predictors identified by univariate analysis were used to construct five models: Logistic Regression (LR), Extreme Gradient Boosting (XGBoost), Decision Tree (DT), Support Vector Machine (SVM), and Random Forest (RF). Model performance was evaluated using the area under the curve (AUC), accuracy, precision, recall, and F1 score. The Shapley Additive Explanations (SHAP) was applied for model interpretation.

**Results:**

Eight variables were identified as key predictors, including puncture duration, catheterized vein, duration and frequency of mechanical ventilation, catheter dwell time, fetal distress, hypoalbuminemia, and antibiotic use within 24 h after birth. Infection rates increased markedly with prolonged puncture duration (6% for < 30 min vs 78% for 30–60 min vs 93% for >60 min) and catheter dwell time (21% for <14 days vs 32% for 14–21 days vs 47% for >21 days). Femoral vein catheterization showed the highest infection rate (82%). In the internal validation set, the AUCs of LR, XGBoost, DT, SVM, and RF were 0.89, 0.86, 0.87, 0.88, and 0.95, respectively; in the external validation set, they were 0.89, 0.88, 0.87, 0.93, and 0.96. The RF model achieved the highest accuracy in both internal (0.91) and external (0.90) validation sets, while SVM showed the highest external accuracy (0.92). Precision ranged from 0.79 to 0.89, recall from 0.27 to 0.31, and F1 scores from 0.42 to 0.45. SHAP analysis showed that puncture duration was the most important predictor, followed by catheterized vein and mechanical ventilation–related variables.

**Conclusions:**

The RF model demonstrated superior performance in predicting CRBSI risk in preterm infants with PICC placement. This model may facilitate early identification of high-risk patients and support clinical decision-making.

## 1 Introduction

With the continuous improvement in the treatment of preterm infants, peripherally inserted central catheters (PICC) have been widely used in this population. PICC offers several advantages, including reducing the need for repeated venipuncture, alleviating pain in infants, and minimizing vascular irritation caused by medications (1). As a result, its utilization has increased year by year (2). Although PICC provides significant convenience in the treatment of preterm infants, long-term catheterization may lead to a series of complications, such as phlebitis, catheter malposition or even accidental dislodgement, difficulty in catheter removal, catheter occlusion, catheter fracture, and catheter-related bloodstream infections (CRBSIs) (3). CRBSI is one of the most serious complications associated with PICC, and once it occurs, it can pose a substantial threat to neonatal health (4). CRBSI not only shortens catheter dwell time but also increases the rate of catheter reinsertion and healthcare costs. Moreover, it is an important factor that can endanger the lives of preterm infants (5). Studies have shown that each episode of CRBSI can increase medical expenses by approximately USD 11,971 to 68,000 (6, 7). A retrospective study reported a mortality rate of 10.26% associated with PICC-related bloodstream infections (8).

Machine learning (ML) can be used to predict the occurrence of diseases (9–12). In recent years, many researchers have reported the advantages of ML in clinical prediction models and applied it to the prediction of various diseases (13, 14). However, machine learning–based risk prediction models for PICC-associated bloodstream infections in preterm infants remain scarce. Therefore, this study aims to develop a machine learning–based risk prediction model for PICC-associated bloodstream infections in preterm infants, in order to provide a basis for identifying high-risk patients and supporting clinical decision-making.

## 2 Materials and methods

### 2.1 Study subjects

This retrospective multicenter study was conducted in five tertiary hospitals in Henan Province, China, including the Third Affiliated Hospital of Zhengzhou University, Xuchang Maternal and Child Health Hospital, Shangqiu First People's Hospital, Zhoukou Central Hospital, and Nanyang Central Hospital. All participating centers have neonatal intensive care units and routinely perform PICC placement, catheter maintenance, and infection surveillance for preterm infants. A total of 151 preterm infants with CRBSI who underwent PICC placement in the Neonatal Department of a tertiary hospital in Henan Province (our hospital) from July 2017 to June 2024 were retrospectively collected. Using a random number table method, a control group was selected at a ratio of 1:2, consisting of 302 preterm infants without CRBSI, matched with the case group by gestational age (±1 week), admission time (±2 weeks), and birth weight (±200 g). The total sample size was 453 cases. Seventy percent of the cases and controls (*n* = 318) were randomly selected to form the training set, while the remaining 30% (*n* = 135) were assigned to the internal validation set. These numbers are derived from a total of 453 eligible preterm infants collected at our hospital.

Additionally, 32 preterm infants with CRBSI who underwent PICC placement in the neonatal departments of four other tertiary hospitals in Henan Province (Xuchang Maternal and Child Health Hospital, Shangqiu First People's Hospital, Zhoukou Central Hospital, and Nanyang Central Hospital) between September 2021 and June 2024 were retrospectively collected. Using the same randomization method, 64 matched controls (1:2 ratio) were selected based on gestational age (±1 week), admission time (±1 month), and birth weight (± 200 g), resulting in a total of 96 cases for external validation.

Inclusion criteria: (1) Preterm infants with gestational age <37 weeks; (2) Infants who underwent PICC placement and maintenance in the neonatal department (all PICC catheters were single-lumen polyurethane catheters). Exclusion criteria: (1) Incomplete medical records; (2) Transfer to another department, treatment withdrawal, or death during catheterization; (3) Presence of systemic infection before catheterization; (4) Multiple catheterizations (≥ 2 times); (5) Presence of other central venous catheters.

This study was approved by the Ethics Committee of the Third Affiliated Hospital of Zhengzhou University (Approval No. 2024-017-01). As a retrospective study, it strictly adhered to the principles of the Declaration of Helsinki and involved only the collection of clinical data, with no actions that could compromise patient privacy. To ensure the protection of personal information and data security, each patient was assigned a unique identification number. Written informed consent was obtained from all patients.

### 2.2 Predictors and outcome measures

Candidate predictors were selected based on literature review, expert consultation, and data availability. A total of 40 variables were ultimately identified, including 12 patient-related factors, 9 disease-related factors, 13 iatrogenic factors, and 6 catheter-related factors. Based on these variables, a “Risk Factor Questionnaire for PICC-Associated Bloodstream Infection in Preterm Infants” was developed. Puncture duration was defined as the total procedural time from the opening of the disinfection package to the completion of dressing application at the puncture site.

To avoid information leakage, the prediction time point in this study was defined as the risk assessment period after PICC insertion and before the occurrence, clinical suspicion, or diagnosis of CRBSI. All candidate predictors used for model development were collected only if they were available before the outcome occurred. Demographic and perinatal variables, including sex, gestational age, birth weight, delivery mode, Apgar score, fetal distress, gestational hypertension, and gestational diabetes, were obtained at birth or admission. Catheterization-related variables, including puncture duration, catheterized vein, type of skin disinfectant, and number of punctures, were recorded at the completion of the PICC procedure. Treatment-related variables, including history of mechanical ventilation, number of mechanical ventilation episodes, duration of mechanical ventilation, history of blood transfusion, and antibiotic use, were counted only up to the time before CRBSI occurrence or the corresponding matched observation time point. Laboratory variables, including white blood cell count, neutrophil count, hemoglobin, and C-reactive protein, were extracted from test results obtained after PICC insertion but before clinical suspicion or diagnosis of CRBSI. Laboratory values obtained at the time of suspected infection, after infection onset, or after CRBSI diagnosis were not used for model development. For non-CRBSI controls, laboratory variables were extracted from results available during PICC catheterization before the corresponding matched observation time point. This approach was used to ensure that all predictors were available before the outcome and to minimize the risk of information leakage.

For infants with suspected CRBSI, peripheral venous blood samples were collected under strict aseptic conditions for blood culture before the initiation or adjustment of antimicrobial therapy whenever clinically feasible. Blood culture samples were processed using the routine automated blood culture systems of the participating centers. Pathogen identification and antimicrobial susceptibility testing were performed by the clinical microbiology laboratories of each participating center according to standardized local laboratory procedures. For each suspected episode, the number of blood culture sets and the volume of blood collected were determined according to the neonatal blood culture protocols of the participating center and the clinical condition of the infant. According to the 2021 Guidelines for the Prevention and Control of Intravascular Catheter-Related Infections, the diagnostic criteria for CRBSI (15) were as follows: the occurrence of bacteremia or fungemia during catheterization or within 48 h after catheter removal, accompanied by clinical manifestations such as fever (≥38 °C), chills, or hypotension, with no other identifiable source of infection. Microbiological criteria included: positive blood cultures for bacteria or fungi, or the isolation of the same pathogen with identical antimicrobial susceptibility results from both catheter tip culture and blood culture.

The incidence of CRBSI was evaluated using two indicators: the crude infection rate and the catheter-day incidence rate. The crude infection rate was calculated as the number of CRBSI cases divided by the total number of preterm infants who underwent PICC placement. The catheter-day incidence rate was calculated as the number of CRBSI cases divided by the total number of PICC catheter-days and expressed per 1,000 catheter-days. The catheter-day incidence rate was automatically generated by the hospital infection surveillance system. Therefore, the crude infection rate reflected the proportion of affected infants among all preterm infants with PICC placement, whereas the catheter-day incidence rate reflected the risk of infection per unit of catheter exposure time.

### 2.3 Sample size calculation

In this study, the sample size was considered from the perspective of clinical prediction model development and machine learning methodology. Although there is currently no universally accepted sample size calculation formula for machine learning-based clinical prediction models, the number of outcome events, the number of candidate predictors, model complexity, and validation strategy are important considerations. A total of 549 preterm infants were included in this study, including 183 infants with CRBSI and 366 matched controls. The primary cohort from our hospital included 453 infants, with 151 CRBSI events, and was used for model development and internal validation. An additional external validation cohort included 96 infants, with 32 CRBSI events.

To reduce the risk of overfitting, the final models were not constructed using all 40 initially collected candidate variables. Instead, univariate analysis was first performed, and eight clinically relevant and statistically significant predictors were selected for model development. Based on 151 CRBSI events in the primary cohort and eight predictors included in the final models, the event-per-variable value was approximately 18.9, which exceeded the commonly used empirical threshold of at least 10 events per predictor variable in prediction model studies (16). In addition, ten-fold cross-validation was used for hyperparameter tuning, and an independent external validation cohort from four additional tertiary hospitals was used to further evaluate model generalizability. Therefore, the sample size was considered acceptable for the development and preliminary validation of the machine learning-based prediction models in this study. Nevertheless, we acknowledge that the sample size, particularly that of the external validation cohort, was still relatively limited. Larger prospective multicenter studies are needed to further validate and optimize the model.

### 2.4 Model development and validation

The valid data collected from our hospital were randomly divided into a training set and an internal validation set at a ratio of 7:3. Specifically, 318 cases were included in the training set, while the remaining 135 cases formed the internal validation set. To enhance the generalizability of the model, an additional 96 cases from external hospitals were incorporated as an external validation set. The training set was used to develop prediction models, while the internal and external validation sets were used to evaluate model performance. Specifically, before model training, key predictors were selected using univariate analysis to simplify the feature set and reduce noise from high-dimensional variables, aiming to balance model interpretability and predictive performance. Five models were then constructed to predict CRBSI in preterm infants: Random Forest (RF), Support Vector Machine (SVM), Extreme Gradient Boosting (XGBoost), Logistic Regression (LR), and Decision Tree (DT). Model performance was compared using the area under the receiver operating characteristic curve (AUC), accuracy, precision, recall, and F1 score to identify the optimal model.

### 2.5 Statistical analysis

Statistical analyses and model development were performed using IBM SPSS version 27.0, SAS version 9.4, R software (version 4.4.2), and Python. Continuous variables were tested for normality. Normally distributed data were presented as mean ± standard deviation (mean ± SD) and compared using the independent samples t-test. Non-normally distributed data were expressed as median with interquartile range and compared using the Mann–Whitney U test. Categorical variables were presented as frequency and percentage, and comparisons between groups were performed using the chi-square test, continuity-corrected chi-square test, or Fisher's exact test, as appropriate.

Univariate analysis was conducted to identify potential risk factors associated with CRBSI in preterm infants. Variables with statistical significance (*P* < 0.05) were selected as candidate predictors for model development. Machine learning models, including LR, XGBoost, DT, SVM, and RF, were constructed using the selected predictors. The dataset from our hospital was randomly divided into a training set and an internal validation set at a ratio of 7:3. An independent external validation cohort from four additional hospitals was used to further assess model generalizability.

Hyperparameters of the models were optimized using ten-fold cross-validation on the training set. Model performance was evaluated using the AUC, accuracy, precision, recall, and F1 score. The occurrence of CRBSI was defined as the positive class. Accuracy, precision, recall, and F1 score were calculated based on the corresponding confusion matrix values, including true positives, false positives, true negatives, and false negatives. Accuracy was defined as the proportion of correctly classified cases among all cases; precision was defined as the proportion of true positive cases among all cases predicted as positive by the model; recall was defined as the proportion of correctly identified CRBSI cases among all true CRBSI cases; and the F1 score was calculated as the harmonic mean of precision and recall. Because this study used a case-control sampling design, with an approximate CRBSI-to-control ratio of 1:2, the proportion of positive cases in the modeling datasets was approximately one-third. Therefore, the subsequently reported model performance metrics reflect the classification performance of the models within the case-control modeling datasets, rather than screening performance calculated based on the natural incidence of CRBSI in the original population of preterm infants with PICC placement. Accuracy was not interpreted as a standalone measure of model performance, but was considered together with AUC, precision, recall, and F1 score.

Receiver operating characteristic (ROC) curves were plotted to assess the discriminative ability of each model in both internal and external validation sets. To improve model interpretability, Shapley Additive Explanations (SHAP) were applied to the optimal model. SHAP values were calculated to quantify the contribution of each predictor and to rank variable importance. All statistical tests were two-sided, and a *P* value < 0.05 was considered statistically significant.

## 3 Results

### 3.1 Study population selection process, incidence, and microbiological findings of CRBSI in preterm infants

In this study, a total of 4,128 neonates who underwent PICC placement in the neonatal department of a tertiary hospital in Henan Province (our hospital) from July 2017 to June 2024 were included. Among them, 156 were full-term neonates (>37 weeks), 3,189 were preterm infants without infection, 15 had umbilical venous catheter–related bloodstream infections, and 17 had incomplete clinical data. Ultimately, 151 preterm infants with CRBSI were identified. During the same period, 302 preterm infants without CRBSI were selected as controls, matched to the case group by gestational age (±1 week), admission time (±2 weeks), and birth weight (±200 g). Among approximately 3,972 preterm infants who underwent PICC placement in our hospital during the study period, 151 CRBSI cases were identified, corresponding to a crude infection rate of approximately 3.8%. In addition, the catheter-day incidence rate automatically generated by the hospital infection surveillance system was 2.5 per 1,000 catheter-days.

Additionally, 685 neonates who underwent PICC placement in the neonatal departments of four other tertiary hospitals in Henan Province (external hospitals) from September 2021 to June 2024 were included. Among them, 33 were full-term neonates (>37 weeks), 609 were preterm infants without infection, 4 had umbilical venous catheter–related bloodstream infections, and 7 had incomplete clinical data. A total of 32 preterm infants with CRBSI were identified. During the same period, 64 matched preterm infants without CRBSI were selected as controls, based on gestational age (±1 week), admission time (±1 month), and birth weight (±200 g). Among approximately 652 preterm infants who underwent PICC placement in the external hospitals during the study period, 32 CRBSI cases were identified, corresponding to a crude infection rate of approximately 4.9%. The catheter-day incidence rate automatically generated by the hospital infection surveillance system was 2.6 per 1,000 catheter-days. Thus, the values of 2.5 and 2.6 per 1,000 catheter-days represented catheter-day incidence rates rather than crude infection rates calculated using the number of infants as the denominator.

A total of 183 pathogenic isolates were identified among infants with CRBSI. Gram-negative bacteria accounted for the largest proportion, with 91 isolates (49.73%), followed by Gram-positive bacteria with 77 isolates (42.08%) and fungi with 15 isolates (8.20%). Among the Gram-negative bacteria, Klebsiella pneumoniae was the most common pathogen, with 51 isolates (27.87%), followed by Serratia marcescens with 17 isolates (9.29%), Enterobacter cloacae with 7 isolates (3.83%), Pseudomonas aeruginosa with 6 isolates (3.28%), Stenotrophomonas maltophilia with 2 isolates (1.09%), Klebsiella ozaenae with 2 isolates (1.09%), Serratia liquefaciens with 2 isolates (1.09%), Escherichia coli with 1 isolate (0.55%), Acinetobacter baumannii with 1 isolate (0.55%), Proteus mirabilis with 1 isolate (0.55%), and Citrobacter freundii with 1 isolate (0.55%). Among the Gram-positive bacteria, Staphylococcus epidermidis was the most frequently isolated pathogen, with 29 isolates (15.85%), followed by Staphylococcus haemolyticus with 12 isolates (6.56%), Enterococcus faecalis with 9 isolates (4.92%), Staphylococcus capitis with 5 isolates (2.73%), Staphylococcus aureus with 4 isolates (2.19%), Staphylococcus warneri with 4 isolates (2.19%), Enterococcus faecium with 4 isolates (2.19%), Bacillus cereus with 3 isolates (1.64%), Staphylococcus hominis with 2 isolates (1.09%), Staphylococcus saprophyticus with 1 isolate (0.55%), Staphylococcus sciuri with 1 isolate (0.55%), aerobic Bacillus with 1 isolate (0.55%), Corynebacterium matruchotii with 1 isolate (0.55%), and Corynebacterium striatum with 1 isolate (0.55%). Among the fungal isolates, Candida albicans and Candida glabrata were the most common, with 4 isolates each (2.19%), followed by Candida tropicalis with 2 isolates (1.09%), Candida lusitaniae with 2 isolates (1.09%), Candida parapsilosis with 2 isolates (1.09%), and Candida krusei with 1 isolate (0.55%).

### 3.2 Baseline characteristics between the training set and the internal validation set

As shown in Table 1, the baseline characteristics between the training set and the internal validation set were generally comparable, with most variables showing no statistically significant differences (*P* > 0.05), indicating good overall balance between the two groups. Among the variables with statistically significant differences, in terms of demographic characteristics, gestational age distribution differed significantly between the two groups (*P* = 0.006), with a higher proportion of infants with gestational age > 32 weeks and a lower proportion of extremely preterm infants (<28 weeks) in the internal validation set. Birth weight distribution also showed a significant difference (*P* = 0.003), with a higher proportion of infants weighing >1.5 kg and a lower proportion of extremely low birth weight infants (<1.0 kg) in the internal validation set. Apgar scores at 1 min (*P* = 0.026) and 5 min (*P* = 0.001) were significantly different between the two groups, with slightly higher scores observed in the internal validation set. Hemoglobin levels also differed significantly (*P* = 0.004), with higher levels in the internal validation set. Regarding perinatal and treatment-related factors, a history of neonatal asphyxia was significantly different between the two groups (*P* = 0.018), with a higher proportion in the training set. The type of skin disinfectant used during catheterization also showed a significant difference (*P* = 0.005), with chlorhexidine more frequently used in the internal validation set. Antibiotic use within 24 h after birth differed significantly (*P* = 0.022), with a higher proportion in the training set. A history of mechanical ventilation was also significantly different (*P* = 0.038), with a higher proportion in the training set. In addition, the number of mechanical ventilation episodes (*P* = 0.044) and the number of blood sampling tests during catheterization (*P* = 0.015) were significantly different between the two groups, both being slightly higher in the training set. All other variables showed no statistically significant differences between the two groups (*P* > 0.05).

**Table 1:** Baseline characteristics of patients in the training cohort and internal validation cohort.

Variable	Category	Training cohort (*N* = 318)	Internal validation cohort (*N* = 135)	P value
Month (%)	Jan–Mar	73 (23.0)	40 (29.6)	0.388
	Apr–Jun	82 (25.8)	27 (20.0)	
	Jul–Sep	98 (30.8)	41 (30.4)	
	Oct–Dec	65 (20.4)	27 (20.0)	
Sex (%)	Male	159 (50.0)	67 (49.6)	0.943
	Female	159 (50.0)	68 (50.4)	
Gestational age (weeks, %)	> 32	23 (7.2)	21 (15.6)	0.006
	28–32	205 (64.5)	89 (65.9)	
	<28	90 (28.3)	25 (18.5)	
Birth weight (kg, %)	> 1.5	14 (4.4)	17 (12.6)	0.003
	1.0–1.5	199 (62.6)	85 (63.0)	
	<1.0	105 (33.0)	33 (24.4)	
Age at catheterization (days, %)	≥7	144 (45.3)	53 (39.3)	0.237
	<7	174 (54.7)	82 (60.7)	
Apgar score at 1 min		8 (6, 9)	8 (7, 9)	0.026
Apgar score at 5 min		9 (8, 9)	9 (8, 10)	0.001
Delivery mode (%)	Vaginal delivery	112 (35.2)	38 (28.1)	0.144
	Cesarean section	206 (64.8)	97 (71.9)	
White blood cell count (×10^9^/L)		8.13 (5.37, 13.19)	8.48 (6.09, 12.42)	0.420
Neutrophil count (×10^9^/L)		27.2 (3.98, 47.5)	28.1 (4.32, 47.2)	0.536
Hemoglobin (g/L)		162 (146, 178)	170 (154, 186)	0.004
C-reactive protein (×10^9^/L)		0.47 (0.2, 0.5)	0.49 (0.2, 0.5)	0.827
Feeding intolerance (%)	Yes	19 (6.0)	9 (6.7)	0.780
Hypoproteinemia (%)	Yes	73 (23.0)	29 (21.5)	0.731
History of neonatal asphyxia (%)	Yes	106 (33.3)	30 (22.2)	0.018
Gestational hypertension (%)	Yes	92 (28.9)	48 (35.6)	0.163
Placental abruption (%)	Yes	4 (1.3)	1 (0.7)	> 0.999
Gestational diabetes (%)	Yes	40 (12.6)	14 (10.4)	0.507
Fetal distress (%)	Yes	136 (42.8)	51 (37.8)	0.324
Premature rupture of membranes (%)	Yes	54 (17.0)	19 (14.1)	0.442
Positive vaginal secretion bacterial culture (%)	Yes	18 (5.7)	6 (4.4)	0.597
Skin disinfectant used (%)	Chlorhexidine	152 (47.8)	84 (62.2)	0.005
	Iodophor	166 (52.2)	51 (37.8)	
Number of punctures	—	1 (1,1)	1 (1,1)	0.673
Puncture duration (%)	<30 min	196 (61.6)	87 (64.4)	0.770
	30–60 min	112 (35.2)	43 (31.9)	
	>60 min	10 (3.1)	5 (3.7)	
Catheterized vein (%)	Basilic vein	63 (19.8)	17 (12.6)	0.269
	Axillary vein	9 (2.8)	7 (5.2)	
	Great saphenous vein	220 (69.2)	98 (72.6)	
	Small saphenous vein	8 (2.5)	3 (2.2)	
	Femoral vein	18 (5.7)	10 (7.4)	
Maintenance by IV therapy team (%)	Yes	72 (22.6)	38 (28.1)	0.211
Antibiotics within 24 h (%)	Yes	253 (79.6)	94 (69.6)	0.022
History of mechanical ventilation (%)	Yes	127 (39.9)	40 (29.6)	0.038
Number of mechanical ventilation episodes (times)	—	0 (0, 1)	0 (0, 1)	0.044
Duration of mechanical ventilation (days)	—	0 (0, 4)	0 (0, 3)	0.068
History of blood transfusion (times)	—	1 (0, 2)	1 (0, 2)	0.165
Number of blood tests during catheterization	—	9 (6, 12)	8 (6, 10)	0.015
Surgical history (%)	Yes	21 (6.6)	8 (5.9)	0.788
Catheter dislocation (%)	Yes	6 (1.9)	0 (0.0)	0.247
Catheter occlusion (%)	Yes	3 (0.9)	2 (1.5)	0.992
Dressing loosening (%)	Yes	2 (0.6)	0 (0.0)	> 0.999
Catheter-related skin injury (%)	Yes	2 (0.6)	1 (0.7)	> 0.999
Catheter indwelling time (days, %)	> 21	108 (34.0)	39 (28.9)	0.571
	14–21	117 (36.8)	54 (40.0)	
	<14	93 (29.2)	42 (31.1)	

### 3.3 Baseline characteristics between the training set and the external validation cohort

As shown in Table 2, most baseline characteristics between the training set and the external validation set were not significantly different (*P* > 0.05), indicating an overall acceptable comparability between the two groups, although several variables showed statistically significant differences. Among the significant variables, in terms of demographic and basic clinical characteristics, birth weight distribution differed significantly between the two groups (*P* < 0.001), with a higher proportion of infants weighing >1.5 kg and a lower proportion of extremely low birth weight infants (<1.0 kg) in the external validation set. The 1-minute Apgar score also showed a significant difference (*P* = 0.034), with slightly lower scores in the external validation set. Regarding laboratory indicators, neutrophil count (*P* < 0.001) and C-reactive protein levels (*P* < 0.001) were significantly higher in the external validation set, suggesting a higher level of inflammatory response. In terms of perinatal and maternal factors, significant differences were observed in neonatal asphyxia (*P* = 0.002), gestational hypertension (*P* = 0.009), and fetal distress (*P* = 0.010), all of which were more prevalent in the training set.

**Table 2:** Baseline characteristics of patients in the training cohort and external validation cohort.

Variable	Category	Training cohort (*N* = 318)	External validation cohort (*N* = 96)	P value
Month (%)	Jan–Mar	73 (23.0)	34 (35.4)	0.063
	Apr–Jun	82 (25.8)	16 (16.7)	
	Jul–Sep	98 (30.8)	29 (30.2)	
	Oct–Dec	65 (20.4)	17 (17.7)	
Sex (%)	Male	159 (50.0)	45 (46.9)	0.591
	Female	159 (50.0)	51 (53.1)	
Gestational age (weeks, %)	>32	23 (7.2)	12 (12.5)	0.074
	28–32	205 (64.5)	66 (68.8)	
	<28	90 (28.3)	18 (18.8)	
Birth weight (kg, %)	>1.5	14 (4.4)	16 (16.7)	<0.001
	1.0–1.5	199 (62.6)	57 (59.4)	
	<1.0	105 (33.0)	23 (24.0)	
Age at catheterization (days, %)	≥7	144 (45.3)	33 (34.4)	0.058
	<7	174 (54.7)	63 (65.6)	
Apgar score at 1 min		8 (6, 9)	7 (5.5, 8)	0.034
Apgar score at 5 min		9 (8, 9)	9 (7, 9)	0.481
Delivery mode (%)	Vaginal delivery	112 (35.2)	34 (35.4)	0.972
	Cesarean section	206 (64.8)	62 (64.6)	
White blood cell count (×10^9^/L)		8.13 (5.37,13.19)	9.415 (6.06,14.045)	0.075
Neutrophil count (×10^9^/L)		27.2 (3.98,47.5)	40.55 (24.97,62.1)	<0.001
Hemoglobin (g/L)		162 (146,178)	168 (152,183)	0.128
C-reactive protein (×10^9^/L)		0.47 (0.2,0.5)	0.5 (0.499,1.39)	<0.001
Feeding intolerance (%)	Yes	19 (6.0)	10 (10.4)	0.135
Hypoproteinemia (%)	Yes	73 (23.0)	23 (24.0)	0.838
History of neonatal asphyxia (%)	Yes	106 (33.3)	16 (16.7)	0.002
Gestational hypertension (%)	Yes	92 (28.9)	15 (15.6)	0.009
Placental abruption (%)	Yes	4 (1.3)	1 (1.0)	>0.999
Gestational diabetes (%)	Yes	40 (12.6)	17 (17.7)	0.201
Fetal distress (%)	Yes	136 (42.8)	27 (28.1)	0.010
Premature rupture of membranes (%)	Yes	54 (17.0)	23 (24.0)	0.124
Positive vaginal secretion bacterial culture (%)	Yes	18 (5.7)	8 (8.3)	0.344
Skin disinfectant used (%)	Chlorhexidine	152 (47.8)	10 (10.4)	<0.001
	Iodophor	166 (52.2)	86 (89.6)	
Number of punctures	—	1 (1,1)	1 (1,1.5)	0.406
Puncture duration (%)	<30 min	196 (61.6)	57 (59.4)	0.013
	30–60 min	112 (35.2)	29 (30.2)	
	>60 min	10 (3.1)	10 (10.4)	
Catheter vein (%)	Basilic vein	63 (19.8)	12 (12.5)	0.262
	Axillary vein	9 (2.8)	1 (1.0)	
	Great saphenous vein	220 (69.2)	73 (76.0)	
	Small saphenous vein	8 (2.5)	5 (5.2)	
	Femoral vein	18 (5.7)	5 (5.2)	
IV team maintenance (%)	Yes	72 (22.6)	37 (38.5)	0.002
Antibiotics within 24 h (%)	Yes	253 (79.6)	68 (70.8)	0.073
Mechanical ventilation history (%)	Yes	127 (39.9)	47 (49.0)	0.117
Number of mechanical ventilation episodes	—	0 (0,1)	0 (0,1)	0.163
Duration of mechanical ventilation (days)	—	0 (0,4)	0 (0,6)	0.096
History of blood transfusion	—	1 (0,2)	2 (1,3)	0.054
Number of blood tests during catheterization	—	9 (6,12)	7 (4,10.5)	0.001
Surgical history (%)	Yes	21 (6.6)	9 (9.4)	0.359
Catheter dislocation (%)	Yes	6 (1.9)	3 (3.1)	0.742
Catheter occlusion (%)	Yes	3 (0.9)	3 (3.1)	0.280
Dressing loosening (%)	Yes	2 (0.6)	0 (0.0)	>0.999
Catheter-related skin injury (%)	Yes	2 (0.6)	0 (0.0)	>0.999
Catheter indwelling time (days, %)	>21	108 (34.0)	33 (34.4)	0.683
	14–21	117 (36.8)	39 (40.6)	
	<14	93 (29.2)	24 (25.0)	

Regarding procedure- and treatment-related variables, the type of skin disinfectant used during catheterization differed significantly (*P* < 0.001), with povidone-iodine predominantly used in the external validation set, while chlorhexidine was more commonly used in the training set. Puncture duration also showed a significant difference (*P* = 0.013), with a higher proportion of procedures lasting >60 min in the external validation set. In addition, daily infusion connection and maintenance performed by an intravenous therapy team were more frequent in the external validation set (*P* = 0.002). The number of blood sampling tests during catheterization was also significantly different (*P* = 0.001), with higher frequencies observed in the training set. All other variables showed no statistically significant differences between the two groups (*P* > 0.05).

### 3.4 Univariate analysis

This study analyzed the potential risk factors for CRBSI in preterm infants in our hospital. The results showed that hypoalbuminemia, fetal distress, puncture duration, catheterized vein, antibiotic use within 24 h after birth, duration of mechanical ventilation, number of mechanical ventilation episodes, and catheter dwell time were significantly associated with infection (*P* < 0.05). Based on these findings, the above variables were selected as key predictors for the construction of the prediction model. Details were shown in Table 3.

**Table 3:** Univariate analysis of patients in our hospital.

Variable	Category	Infection (*N* = 151)	Non-infection (*N* = 302)	P value
Month (%)	Jan–Mar	38 (25.2)	75 (24.8)	0.803
	Apr–Jun	38 (25.2)	71 (23.5)	
	Jul–Sep	42 (27.8)	97 (32.1)	
	Oct–Dec	33 (21.9)	59 (19.5)	
Sex (%)	Male	79 (52.3)	147 (48.7)	0.465
	Female	72 (47.7)	155 (51.3)	
Gestational age (weeks, %)	>32	14 (9.3)	30 (9.9)	0.565
	28–32	94 (62.3)	200 (66.2)	
	<28	43 (28.5)	72 (23.8)	
Birth weight (kg, %)	>1.5	8 (5.3)	23 (7.6)	0.184
	1.0–1.5	89 (58.9)	195 (64.6)	
	<1.0	54 (35.8)	84 (27.8)	
Age at catheterization (days, %)	≥7	71 (47.0)	126 (41.7)	0.284
	<7	80 (53.0)	176 (58.3)	
Apgar score at 1 min		8 (6,9)	8 (7,9)	0.162
Apgar score at 5 min		9 (8,9)	9 (8,9)	0.197
Delivery mode (%)	Vaginal delivery	46 (30.5)	104 (34.4)	0.397
	Cesarean section	105 (69.5)	198 (65.6)	
White blood cell count (×10^9/L)		8.44 (5.58,13.14)	8.095 (5.47,12.93)	0.575
Neutrophil count (×10^9/L)		28.4 (4.32,50.1)	26.5 (3.98,47.2)	0.303
Hemoglobin (g/L)		163.03 ± 22.82	164.33 ± 24.62	0.587
C-reactive protein (×10^9/L)		0.42 (0.2,0.5)	0.49 (0.2,0.51)	0.247
Feeding intolerance (%)	Yes	10 (6.6)	18 (6.0)	0.783
Hypoproteinemia (%)	Yes	52 (34.4)	50 (16.6)	<0.001
History of neonatal asphyxia (%)	Yes	37 (24.5)	99 (32.8)	0.070
Gestational hypertension (%)	Yes	46 (30.5)	94 (31.1)	0.886
Placental abruption (%)	Yes	1 (0.7)	4 (1.3)	0.874
Gestational diabetes (%)	Yes	22 (14.6)	32 (10.6)	0.219
Fetal distress (%)	Yes	81 (53.6)	106 (35.1)	<0.001
Premature rupture of membranes (%)	Yes	26 (17.2)	47 (15.6)	0.651
Positive vaginal secretion bacterial culture (%)	Yes	6 (4.0)	18 (6.0)	0.374
Skin disinfectant used (%)	Chlorhexidine	77 (51.0)	159 (52.6)	0.740
	Iodophor	74 (49.0)	143 (47.4)	
Number of punctures	—	1 (1,1)	1 (1,1)	0.807
Puncture duration (%)	<30 min	16 (10.6)	267 (88.4)	<0.001
	30–60 min	121 (80.1)	34 (11.3)	
	>60 min	14 (9.3)	1 (0.3)	
Catheter vein (%)	Basilic vein	27 (17.9)	53 (17.5)	<0.001
	Axillary vein	3 (2.0)	13 (4.3)	
	Great saphenous vein	96 (63.6)	222 (73.5)	
	Small saphenous vein	2 (1.3)	9 (3.0)	
	Femoral vein	23 (15.2)	5 (1.7)	
IV team maintenance (%)	Yes	39 (25.8)	71 (23.5)	0.588
Antibiotics within 24 h (%)	Yes	125 (82.8)	222 (73.5)	0.028
Mechanical ventilation history (%)	Yes	63 (41.7)	104 (34.4)	0.130
Number of mechanical ventilation episodes	—	0 (0,1)	0 (0,1)	0.018
Duration of mechanical ventilation (days)	—	0 (0,5)	0 (0,3)	0.025
History of blood transfusion	—	1 (0,2)	1 (0,2)	0.939
Number of blood tests during catheterization	—	8 (6,11)	8 (6,11)	0.277
Surgical history (%)	Yes	12 (7.9)	17 (5.6)	0.342
Catheter dislocation (%)	Yes	3 (2.0)	3 (1.0)	0.663
Catheter occlusion (%)	Yes	2 (1.3)	3 (1.0)	>0.999
Dressing loosening (%)	Yes	2 (1.3)	0 (0.0)	0.111
Catheter-related skin injury (%)	Yes	2 (1.3)	1 (0.3)	0.539
Catheter indwelling time (days, %)	>21	69 (45.7)	78 (25.8)	<0.001
	14–21	54 (35.8)	117 (38.7)	
	<14	28 (18.5)	107 (35.4)	

### 3.5 Distribution of categorical predictors for CRBSI in training and external validation sets

As shown in Table 4, in the data from our hospital, several categorical variables were clearly associated with the occurrence of CRBSI in preterm infants. First, puncture duration showed a strong positive relationship with infection risk: the infection rate was only 6% when the duration was <30 min, but increased markedly to 78% and 93% for 30–60 min and > 60 min, respectively, indicating that longer puncture time is associated with higher infection risk. Second, catheterized vein had a notable impact, with the highest infection rate observed in the femoral vein (82%), which was substantially higher than that in the basilic vein (34%) and great saphenous vein (30%). Third, catheter dwell time demonstrated an increasing trend in infection risk with prolonged duration, with infection rates of 47% for >21 days, 32% for 14–21 days, and 21% for <14 days. In addition, infants with fetal distress (43% vs 26%) and hypoalbuminemia (51% vs 28%) had significantly higher infection rates compared to those without these conditions. The infection rate was also higher among those who received antibiotics within 24 h after birth (36% vs 25%), suggesting a potential association with increased infection risk.

**Table 4:** Distribution of predictive factors (categorical variables) for CRBSI in preterm infants with PICC in our hospital.

Variable	Category	Total (N)	Non-infection (%)	Infection (%)
Puncture duration	<30 min	283	267 (94)	16 (6)
	30–60 min	155	34 (22)	121 (78)
	>60 min	15	1 (7)	14 (93)
Catheterized vein	Basilic vein	80	53 (66)	27 (34)
	Axillary vein	16	13 (81)	3 (19)
	Great saphenous vein	318	222 (70)	96 (30)
	Small saphenous vein	11	9 (82)	2 (18)
	Femoral vein	28	5 (18)	23 (82)
Catheter indwelling time (days)	>21	147	78 (53)	69 (47)
	14–21	171	117 (68)	54 (32)
	<14	135	107 (79)	28 (21)
Fetal distress	Yes	187	106 (57)	81 (43)
	No	266	196 (74)	70 (26)
Hypoproteinemia	Yes	102	50 (49)	52 (51)
	No	351	252 (72)	99 (28)
Antibiotics within 24 h after birth	Yes	347	222 (64)	125 (36)
	No	106	80 (75)	26 (25)

As shown in Table 5, similar patterns were observed in the external validation data. Puncture duration remained positively associated with infection risk (4%, 72%, and 90% for <30 min, 30–60 min, and >60 min, respectively). For catheterized vein, the femoral vein again showed the highest infection rate (80%), while no infection cases were observed in the axillary vein or small saphenous vein groups. Catheter dwell time also demonstrated a consistent increasing trend (52% for >21 days, 26% for 14–21 days, and 21% for <14 days). Furthermore, both fetal distress (67% vs 20%) and hypoalbuminemia (65% vs 23%) were associated with substantially higher infection rates. Regarding antibiotic use within 24 h after birth, the infection rate was 47% among those who received antibiotics, whereas no infections were observed in those who did not (0%), again suggesting a potential association with infection risk.

**Table 5:** Distribution of predictive factors (categorical variables) for CRBSI in preterm infants with PICC outside our hospital.

Variable	Category	Total (N)	Non-infection (%)	Infection (%)
Puncture duration	<30 min	57	55 (96)	2 (4)
	30–60 min	29	8 (28)	21 (72)
	>60 min	10	1 (10)	9 (90)
Catheterized vein	Basilic vein	12	7 (58)	5 (42)
	Axillary vein	1	1 (100)	0 (0)
	Great saphenous vein	73	50 (68)	23 (32)
	Small saphenous vein	5	5 (100)	0 (0)
	Femoral vein	5	1 (20)	4 (80)
Catheter indwelling time (days)	>21	33	16 (48)	17 (52)
	14–21	39	29 (74)	10 (26)
	<14	24	19 (79)	5 (21)
Fetal distress	Yes	27	9 (33)	18 (67)
	No	69	55 (80)	14 (20)
Hypoproteinemia	Yes	23	8 (35)	15 (65)
	No	73	56 (77)	17 (23)
Antibiotics within 24 h after birth	Yes	68	36 (53)	32 (47)
	No	28	28 (100)	0 (0)

### 3.6 Model development

Based on the results of the univariate analysis, hypoalbuminemia, fetal distress, puncture duration, catheterized vein, antibiotic use within 24 h after birth, duration of mechanical ventilation, number of mechanical ventilation episodes, and catheter dwell time were significantly associated with infection (*P* < 0.05). These variables were therefore selected as key predictors for model development.

The dataset from our hospital was randomly divided into a training set and an internal validation set at a ratio of 7:3, including 317 cases in the training set and 136 cases in the internal validation set. In addition, 96 cases from external hospitals were used as an external validation set to evaluate the predictive performance and generalizability of the models. The parameter tuning and final selection of each algorithm based on ten-fold cross-validation were presented in Table 6.

**Table 6:** Parameter selection of models using 10-fold cross-validation for each algorithm.

Model	Code	Parameter range	Final selected parameter
XGBoost			
	eta	0.001–0.1	0.1
	max_depth	1–10	2
	subsample	0.5	0.5
	colsample_bytree	1	1
	gamma	0.1–1	0.1
	min_child_weight	1	1
	nrounds	50–200	100
Decision Tree			
	minsplit	0–100	20
	maxdepth	0–100	30
	cp	0.001–1	0.01
SVM	sigma	0.1–2	1
	C	0.1–2	0.1
Random Forest			
	ntree	10000	10000
	mtry	1–10	2

### 3.7 Model evaluation

The performance of five machine learning models—LR, XGBoost, DT, SVM, and RF—was evaluated using AUC, accuracy, precision, recall, and F1 score (Table 7). These metrics were derived from the confusion matrix of each model, with CRBSI defined as the positive class. Because the modeling cohorts were constructed using case-control sampling rather than the natural incidence distribution of the original PICC population, the accuracy, precision, recall, and F1 score reported in Table 7 should be interpreted as classification metrics within the training, internal validation, and external validation datasets. They should not be directly compared mathematically with the crude infection rate or catheter-day incidence rate in the original population. As shown in Table 7, all five models demonstrated good predictive performance across the training, internal validation, and external validation sets. In the internal validation set, the AUC values of LR, XGBoost, DT, SVM, and RF were 0.89, 0.86, 0.87, 0.88, and 0.95, respectively. In the external validation set, the corresponding AUC values were 0.89, 0.88, 0.87, 0.93, and 0.96. Among them, the RF model showed the highest AUC in both the internal and external validation sets, while XGBoost had the lowest AUC in the internal validation set and DT had the lowest AUC in the external validation set.

**Table 7:** Evaluation of model predictive performance.

Model	Dataset	AUC	Accuracy	Precision	Recall	F1-score
Logistic Regression (LR)	Training set	0.94	0.87	0.81	0.25	0.38
	Internal validation set	0.89	0.90	0.89	0.30	0.45
	External validation set	0.89	0.90	0.82	0.29	0.43
XGBoost	Training set	0.90	0.91	0.87	0.27	0.41
	Internal validation set	0.86	0.87	0.84	0.30	0.43
	External validation set	0.88	0.88	0.79	0.28	0.42
Decision Tree	Training set	0.87	0.89	0.84	0.26	0.39
	Internal validation set	0.87	0.88	0.84	0.30	0.43
	External validation set	0.87	0.88	0.79	0.28	0.42
Support Vector Machine (SVM)	Training set	0.96	0.90	0.82	0.31	0.45
	Internal validation set	0.88	0.87	0.86	0.27	0.42
	External validation set	0.93	0.92	0.80	0.31	0.45
Random Forest (RF)	Training set	0.97	0.92	0.89	0.29	0.44
	Internal validation set	0.95	0.91	0.87	0.29	0.43
	External validation set	0.96	0.90	0.82	0.29	0.43

CRBSI was defined as the positive class. Accuracy, precision, recall, and F1 score were calculated from the corresponding confusion matrix values, including true positives, false positives, true negatives, and false negatives. The metrics shown in this table were calculated within the case-control modeling datasets, in which the ratio of CRBSI cases to non-CRBSI controls was approximately 1:2; therefore, these values do not represent model performance calculated according to the natural CRBSI incidence in the original PICC population.

In terms of accuracy, the RF model achieved the highest values in both the internal validation set (0.91) and the external validation set (0.90). The SVM model showed relatively high accuracy in the external validation set (0.92). The accuracy of the LR, XGBoost, and DT models ranged from 0.87 to 0.90 across datasets. However, accuracy alone was not considered sufficient to evaluate model performance, particularly in the presence of unequal class distribution. Therefore, accuracy was interpreted in combination with AUC, precision, recall, and F1 score.

Regarding precision, all models showed relatively high values, ranging from 0.79 to 0.89 in the validation sets. However, recall values were relatively low across models, ranging from 0.27 to 0.31. The F1 scores ranged from 0.42 to 0.45, indicating moderate overall performance. These results suggest that although the models showed good overall discrimination and relatively high positive predictive accuracy, their ability to identify all true CRBSI cases was limited. Therefore, the models should be used as supportive risk assessment tools rather than as standalone screening tools in clinical practice.

**Figure 1:**
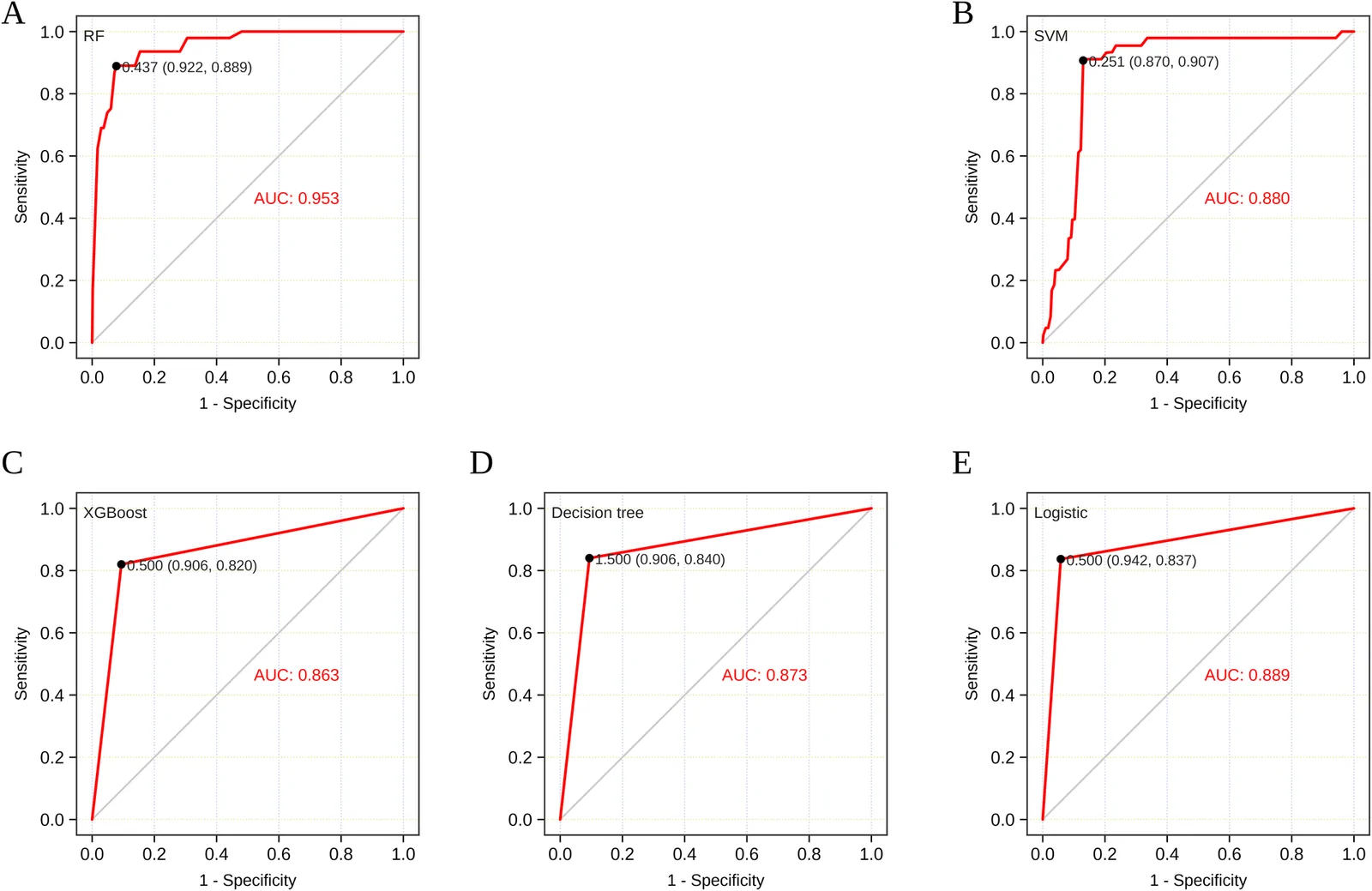
Receiver operating characteristic curves of five machine learning models in the internal validation set. **(A)** Random Forest (RF); **(B)** Support Vector Machine (SVM); **(C)** Extreme Gradient Boosting (XGBoost); **(D)** Decision Tree (DT); **(E)** Logistic Regression (LR). The area under the curve (AUC) values for the five models were 0.953, 0.880, 0.863, 0.873, and 0.889, respectively.

As shown in Figure 1, the ROC curves of the five models in the internal validation set were all above the reference line, indicating good discriminative ability. The RF model had the largest area under the curve (AUC = 0.953), followed by LR (AUC = 0.889), SVM (AUC = 0.880), DT (AUC = 0.873), and XGBoost (AUC = 0.863).

**Figure 2:**
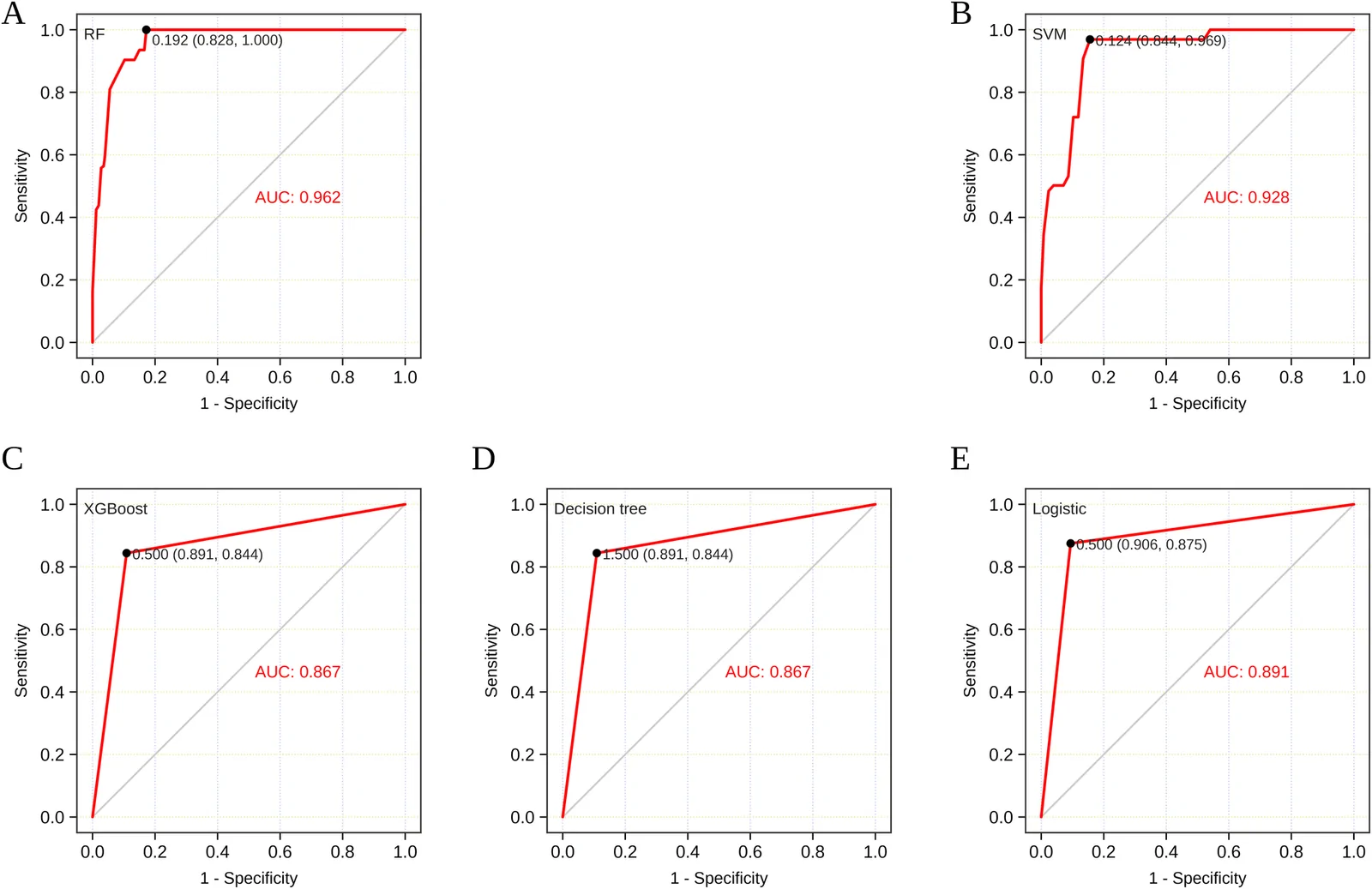
Receiver operating characteristic curves of five machine learning models in the external validation set. **(A)** Random Forest (RF); **(B)** Support Vector Machine (SVM); **(C)** Extreme Gradient Boosting (XGBoost); **(D)** Decision Tree (DT); **(E)** Logistic Regression (LR). The area under the curve (AUC) values for the five models were 0.962, 0.928, 0.867, 0.867, and 0.891, respectively.

As shown in Figure 2, similar patterns were observed in the external validation set. The RF model had the highest AUC (0.962), followed by SVM (0.928), LR (0.891), XGBoost (0.867), and DT (0.867). All ROC curves were located above the diagonal line, indicating acceptable predictive performance across models.

### 3.8 SHAP analysis

**Figure 3:**
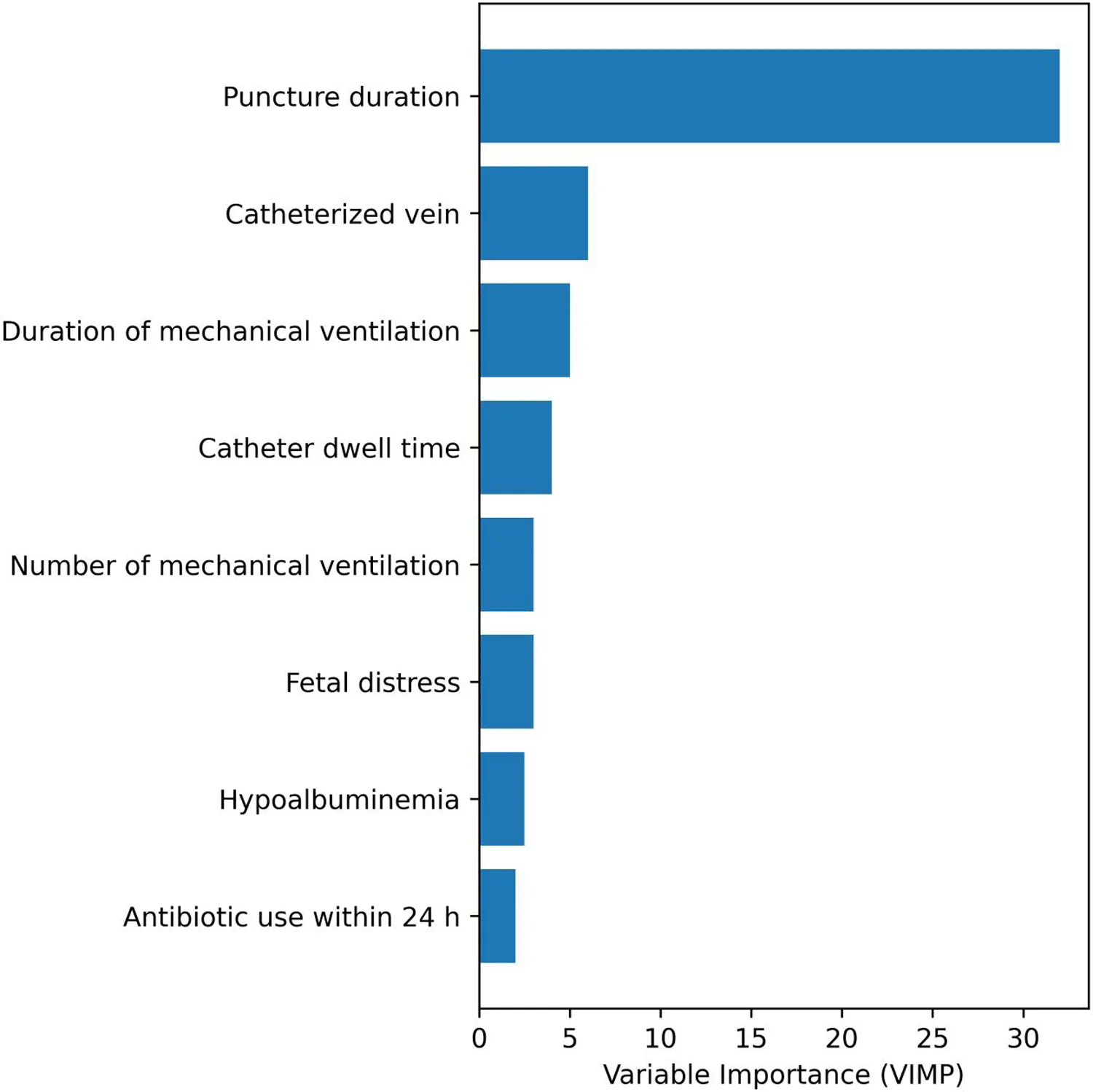
Variable importance ranking in the random forest model based on SHAP values. Variables are ranked according to their contribution to the prediction of CRBSI in preterm infants. Puncture duration showed the highest importance, followed by catheterized vein, duration of mechanical ventilation, catheter dwell time, number of mechanical ventilation episodes, fetal distress, hypoalbuminemia, and antibiotic use within 24 h after birth.

To further explore the key influencing factors of CRBSI in preterm infants and enhance the interpretability of the model, the SHAP method was applied for variable analysis. After dataset partitioning, SHAP values of the RF model were calculated using the Python SHAP library. As shown in Figure 3, the importance of variables in the RF model is ranked according to their SHAP values, with higher importance corresponding to higher positions in the plot. The results indicate that puncture duration had the highest importance, followed by catheterized vein, duration of mechanical ventilation, catheter dwell time, number of mechanical ventilation episodes, fetal distress, hypoalbuminemia, and antibiotic use within 24 h after birth.

## 4 Discussion

CRBSI poses a major challenge to the safety and clinical management of preterm infants and remains an important issue in healthcare quality control. Accurate assessment and prediction of CRBSI in preterm infants are still challenging due to the complex interplay of disease-related, iatrogenic, and catheter-related factors. Therefore, this study aimed to develop and compare five machine learning models that incorporate complex clinical conditions, in order to identify the optimal algorithm for predicting CRBSI risk in preterm infants. The goal was to achieve individualized and precise prediction of infection risk. This study demonstrated that the RF model achieved superior performance in predicting CRBSI risk in preterm infants. Key predictors identified included puncture duration, catheterized vein, mechanical ventilation–related factors (duration and number of episodes), catheter dwell time, fetal distress, hypoalbuminemia, and antibiotic use within 24 h after birth. These findings highlight that longer puncture time, femoral vein catheterization, prolonged mechanical ventilation, and prolonged catheter dwell time significantly increase CRBSI risk, while systemic conditions such as fetal distress and hypoalbuminemia also contribute to susceptibility. The results provide strong clinical relevance by identifying high-risk patients early, enabling targeted preventive measures such as optimizing puncture technique, selecting safer catheter veins, closely monitoring mechanically ventilated infants, and adjusting early antibiotic administration. This emphasizes that predictive modeling can support individualized clinical decision-making and improve neonatal care quality.

In this study, five algorithms—LR, XGBoost, Decision Tree, SVM, and RF—were applied to construct a risk prediction model for CRBSI in preterm infants. Through comprehensive analysis and comparison, the RF model demonstrated the best predictive performance among all models, achieving the highest accuracy and AUC values. These findings provide a reliable basis for early and precise clinical screening of CRBSI risk in preterm infants with PICC placement. In addition, Hairui et al. (17) developed frailty risk prediction models for elderly patients with chronic heart failure (CHF) using LR, DT, SVM, and RF algorithms. Their comparative analysis showed that the RF model ranked first across multiple key indicators, including sensitivity, specificity, accuracy, precision, F1 score, and AUC. The superior performance of the RF model may be attributed to its strong capability in handling high-dimensional data without relying on traditional feature selection methods (18), thereby enhancing its flexibility and adaptability. Moreover, RF has strong robustness against noise and can maintain high predictive accuracy even in the presence of imbalanced data (19), ensuring model stability and reliability. Furthermore, the SHAP analysis in this study identified puncture duration as the most significant predictor of CRBSI. This finding has important practical implications: prolonged puncture time may increase the risk of infection, suggesting that clinical protocols should emphasize minimizing the duration and number of puncture attempts through skilled operators, adequate pre-procedure preparation, and optimized workflow. While specific thresholds for puncture duration need to be validated in different clinical settings, this evidence highlights the potential for targeted procedural improvements to proactively reduce CRBSI risk in preterm infants.

However, this study has several limitations. First, the sample size was relatively small, which may limit the statistical power and generalizability of the findings. Although external validation was performed using data from four additional tertiary hospitals, future studies should include larger, multi-center, and prospective datasets to further validate and generalize the model. Second, some potentially important clinical predictors could not be included in the model due to the retrospective design. These include dressing change frequency, type of infusion connector, and other catheter maintenance practices, which have been reported to influence CRBSI risk. Their absence may affect the relative importance of the predictors identified, and future studies should incorporate these variables to improve model comprehensiveness and predictive accuracy. Third, inflammatory markers such as procalcitonin (PCT) were not collected in this study, and although C-reactive protein was measured, it did not show significant predictive value for CRBSI in our cohort. Future prospective studies should consider including both PCT and C-reactive protein to evaluate their potential roles in early detection of CRBSI and in enhancing predictive model performance. Fourth, in this study, the RF model demonstrated high predictive performance in both internal and external validation, with overall accuracy also being relatively high. However, the recall (sensitivity) of all models remained relatively low. In the context of clinical early-warning applications, a low recall implies that some potential infection cases may be missed. This phenomenon reflects the inherent trade-off between accuracy and sensitivity: while the model maximizes overall accuracy, it may sacrifice part of its recall. In practical application, this indicates that the model should be used in conjunction with clinical judgment and risk management. For example, the classification threshold could be adjusted according to bedside safety requirements to increase sensitivity and minimize missed cases, although this may lead to a higher false-positive rate. Therefore, although the model performs well in predicting CRBSI risk, careful evaluation of the balance between sensitivity and accuracy is necessary when applying it clinically, and decisions should be made in combination with clinical expertise. Fifth, model calibration was not assessed in this study. Because this study adopted a case-control design, the proportion of CRBSI cases in the modeling datasets was determined by sampling and could not represent the true clinical incidence of CRBSI among all preterm infants with PICC placement. In the absence of the true baseline incidence in the target population, sampling weight correction, or model recalibration, directly reporting calibration plots, calibration intercept and slope, Brier score, or Hosmer-Lemeshow statistics may produce misleading conclusions regarding absolute risk calibration. Therefore, the current model should be interpreted primarily as a risk stratification and case classification tool, rather than as a clinical calculator that directly provides calibrated individual absolute risk probabilities. Future prospective studies based on consecutively enrolled real-world cohorts should incorporate the true CRBSI incidence in the target population, perform model recalibration, and further report calibration plots, calibration intercept and slope, Brier score, and Hosmer-Lemeshow statistics to more comprehensively evaluate clinical utility. Sixth, additional performance metrics such as specificity, positive predictive value, negative predictive value, and the area under the precision-recall curve were not reported in this study. Because the modeling datasets were constructed using a fixed case-control sampling ratio, the event proportion did not reflect the true CRBSI incidence in the target clinical population. Positive predictive value, negative predictive value, and the area under the precision-recall curve are strongly affected by event prevalence or the proportion of positive cases; therefore, directly reporting these metrics based on the current case-control sample may overestimate model performance in a real low-incidence clinical setting and may limit the generalizability of the results. Although specificity is not directly dependent on prevalence, it is a threshold-dependent metric, and this study has not yet established a clinically meaningful intervention threshold for initiating intensified monitoring or preventive measures. In contrast, the ROC curve and AUC summarize the trade-off between sensitivity and specificity across all possible thresholds. Therefore, to avoid potentially misleading threshold-dependent or incidence-dependent interpretations, these additional metrics were not added at this stage. Future prospective studies based on consecutively enrolled real-world cohorts should further report specificity, positive predictive value, negative predictive value, and the area under the precision-recall curve after determining the true baseline incidence of CRBSI and clinically meaningful intervention thresholds. Seventh, although antimicrobial susceptibility testing was performed for clinically relevant isolates to guide anti-infective treatment, this multicenter retrospective study included data from different hospitals, and the antimicrobial panels, laboratory platforms, and reporting formats were not completely uniform across centers. Therefore, a unified quantitative analysis of antimicrobial susceptibility patterns for all isolated pathogens could not be performed. Future prospective multicenter studies should standardize blood culture sampling, pathogen identification, and antimicrobial susceptibility testing procedures to further clarify the antimicrobial resistance characteristics of pathogens causing PICC-associated bloodstream infections in preterm infants.

## 5 Conclusions

This study developed a machine learning–based risk prediction model for CRBSI in preterm infants. Among the five models, the RF model showed the best predictive performance. Key predictors included puncture duration, catheterized vein, mechanical ventilation–related factors, catheter dwell time, fetal distress, hypoalbuminemia, and antibiotic use. The model can assist in early identification of high-risk patients and support clinical decision-making. Future studies should include larger, multicenter prospective data and incorporate additional relevant variables to further improve model performance and generalizability.

## Data Availability

The raw data supporting the conclusions of this article will be made available by the authors, without undue reservation.
